# Handling uncertainty in cost-effectiveness analysis in dental medicine: a systematic review with a focus on affordability and risk-aversion

**DOI:** 10.1186/s12962-025-00641-9

**Published:** 2025-06-19

**Authors:** Pedram Sendi, Mojdeh Walterscheidt, Michael M. Bornstein

**Affiliations:** 1 Department of Oral Health & Medicine, University Center of Dental Medicine Basel UZB, Mattenstrasse 40, Basel, 4058 Switzerland; 2 Division of Clinical Epidemiology, Department of Clinical Research, Basel University Hospital, Basel, Switzerland

**Keywords:** Cost-effectiveness, Uncertainty, Dental medicine, Dentistry, Budget impact, Risk aversion, Economic evaluation

## Abstract

**Background:**

The number of published cost-effectiveness analyses in dental medicine has substantially increased in recent years. A key methodological issue in these analyses is how to address uncertainty in costs and effects, which also impacts uncertainty around the expected cost-effectiveness ratio. The cost-effectiveness acceptability curve has become the standard method to summarize uncertainty in probabilistic cost-effectiveness analyses. However, it does not inform decision-makers about budget impact or account for the fact that they are often risk-averse. The cost-effectiveness affordability curve and the cost-effectiveness risk-aversion curve have been proposed to address these limitations. In this systematic review, we assess how uncertainty has been handled in cost-effectiveness analyses in dental medicine, with a particular focus on affordability and risk-aversion.

**Methods:**

We conducted a systematic literature search across electronic databases (MEDLINE, Web of Science, Cochrane Library, EconLit, Embase) on April 18, 2025, and performed manual searches of selected references. Articles published after January 1, 2021, were included. From each study, we extracted information on the first author, year of publication, country, intervention evaluated, study design (model-based, trial-based, or combined), use of deterministic and/or probabilistic sensitivity analysis, and whether budget impact and risk-aversion were considered.

**Results:**

From a total of 57 published cost-effectiveness analyses, 49 (85%) used a deterministic sensitivity analysis and 37 (65%) used a probabilistic sensitivity analysis. Budget impact analysis was performed in five studies (9%), and only one study formally applied both the cost-effectiveness affordability curve and the cost-effectiveness risk-aversion curve.

**Conclusion:**

The use of methods to address uncertainty related to budget constraints and risk-aversion remains limited in dental medicine. As decision-makers often operate within budget constraints and health is considered the most valuable good, incorporating methods that address affordability and risk-aversion could enhance the relevance and impact of cost-effectiveness analyses in dental care.

**Supplementary Information:**

The online version contains supplementary material available at 10.1186/s12962-025-00641-9.

## Introduction

The number of published cost-effectiveness analyses in dental medicine has substantially increased in recent years [1–3] A key methodological challenge in these analyses is how to address uncertainty in costs and effects, which directly impacts the uncertainty surrounding the expected cost-effectiveness ratio [4–7]. A cost-effectiveness analysis may be conducted alongside a clinical trial where cost data are collected in addition to clinical data, or alternatively a cost-effectiveness analysis may be based on a mathematical or decision analytic model where model input parameters may be subject to uncertainty [4, 5, 8, 9]. In a deterministic cost-effectiveness analysis model input parameter or multiple parameters are varied one at a time and their impact on the incremental cost-effectiveness ratio is evaluated [10]. In a probabilistic cost-effectiveness analysis, the joint impact of all parameters subject to uncertainty are evaluated simultaneously using simulation techniques which usually results in a joint distribution of incremental costs and effects that is typically displayed on the cost-effectiveness plane [5, 11–13]. The cost-effectiveness plane is a graphical representation of the incremental costs on the y-axis versus incremental effects on the x-axis [14].

Since the introduction of the cost-effectiveness acceptability curve (CEAC) by van Hout et al. [4], this method to summarize uncertainty around costs and effects has become the standard method to handle uncertainty in probabilistic cost-effectiveness analysis [5, 15]. The CEAC summarizes the joint distribution of incremental costs and effects on the cost-effectiveness plane. The CEAC displays the probability that an intervention is cost-effective for all possible threshold cost-effectiveness ratios (or ceiling ratios), which reflects the decision maker’s maximum willingness to pay (WTP) for an incremental health gain [5, 16, 17]. Although in the absence of uncertainty the threshold cost-effectiveness ratio represents the shadow price of the constrained budget [18, 19], assuming constant returns to scale and complete divisibility of health care programs, in the presence of uncertainty this interpretation becomes less straightforward. This is because a constrained optimization approach to maximizing health subject to a budget constraint does not lead to a threshold cost-effectiveness ratio as a cut-off point for resource allocation [20–22]. Since the advent of the CEAC, additional approaches to summarizing uncertainty around costs and effects have been suggested in the literature that extend the information provided by the CEAC [16, 23, 24].

Since the CEAC does not necessarily inform the decision-maker whether an eventual investment is within the available budget, the cost-effectiveness affordability curve (CEAFC) has been suggested as a method to assess the joint probability that an intervention is both cost-effective and affordable [16]. Furthermore, it has been documented that decision makers are usually risk averse towards large investments and health [22, 23, 25–29]. Since health is regarded as the most valuable good of all, it is natural to be risk-averse towards health [29]. Different methods have been suggested to include risk-aversion into cost-effectiveness analysis [25, 26, 28, 30] but the most pragmatic and straightforward approach is the cost-effectiveness risk-aversion curve (CERAC), which is derived from risk-adjusted performance measures commonly used in finance [23, 31]. Risk-adjusted performance measures assess the return of an investment relative to its associated risk or volatility [31]. The CERAC can be used for any probabilistic cost-effectiveness analysis without requiring additional information [23]. However, there is a lack of evidence on whether these more recent developments have found their way in handling uncertainty in cost-effectiveness analysis in dental medicine.

In the present paper we therefore systematically review the recent literature on the cost-effectiveness of dental interventions and assess whether affordability and risk-aversion have been considered. The present systematic review should therefore shed light on the current status quo with respect to handling uncertainty in cost-effectiveness analysis with a focus on affordability and risk-aversion and may help researchers to consider additional analyses when conducting a cost-effectiveness analysis.

## Methods

The databases MEDLINE via PubMed and Web of Science, EconLit and Embase were searched on April 18th 2025 using a combination of Medical Subject Headings and general search terms. The details of the search strategy and combination of search terms are shown in the Appendix. We limited our search strategy to cost-effectiveness analyses in dental medicine published after January 1st 2021 to account for the fact that the CERAC was presented in 2021 and thereafter [23, 24, 26]. In addition, we conducted reference searches of articles that were identified as reviews. We also searched the Cochrane Library (www.cochranelibrary.com, accessed April 18th, 2025) and the PROSPERO database (www.crd.york.ac.uk/prospero, accessed April 18th, 2025) for published or eventual ongoing studies that assessed the methodology of assessing uncertainty in cost-effectiveness analysis in dental medicine. The present systematic review was not registered on PROSPERO as it focuses on a methodological/econometric topic in cost-effectiveness analysis without a direct effect on health in humans, which is needed for inclusion on PROSPERO (https://www.crd.york.ac.uk/PROSPERO/help/eligibility).

After exclusion of duplicates, titles and abstracts were screened by two reviewers (M.W. and P.S.) and further considered using the following eligibility criteria: publication in English or German and empirical studies evaluating the cost-effectiveness of a dental intervention; If from the abstract and titles eligibility was not clear, the full-text article was screened to assess eligibility. For each included article, we extracted the following study characteristics: first author, year of publication, country, dental good or service provided, study design (model or trial based or combined), and whether a deterministic and/or probabilistic sensitivity analysis was conducted, and whether budget impact and risk aversion were considered in the analysis. Further study characteristics such as intervention/topic, strategies evaluated, time horizon, perspective, discount rate, clinical outcome, cost-effectiveness results and currency/year were also extracted. In addition, the CHEERS 2022 checklist [32] was also elicited and presented as a supplementary file in the Appendix. The included papers were read by two independent reviewers to reduce extraction errors. To illustrate the methodologies of the CEAFC and CERAC, a short tutorial has also been included in the next section, since researchers conducting cost-effectiveness analyses in dentistry may not be familiar with these methodologies. A more detailed explanation has been published previously in medicine using the example of breast cancer treatment [24].

### The CEAFC and CERAC – a primer

 The CEAFC is a modification of the CEAC where the joint probability of an intervention being both cost-effective and affordable is assessed [16, 24]. The CERAC makes use of risk-adjusted performance measures developed in finance, in particular the Sortino ratio, to include risk-aversion into the analysis [23, 24]. To better understand how the CEAFC and CERAC are constructed we may want to use a hypothetical example for demonstration purposes. Consider the following two health care programs (program E and F) as shown in Table 1. The example is similar to a previously published educational article, with more detailed technical explanations [24] Program E has expected costs per patient of $51,000 and mean effects of 11 Quality-Adjusted Life-Years (QALY). And program F has an expected cost of $91,000 and expected health outcomes of 14 QALYs. The respective standard deviations and correlation between costs and effects are also sown in Table 1.

**Table 1:** Costs and effects of two hypothetical programs

Program	µ _C_ ($)	σ _C_ ($)	µ_E_ (QALY)	σ_E_ (QALY)	ρ
E	51,000	5100	11	1.4	0.4
F	91,000	15,000	14	1.2	0.8

µ_C_ denotes mean costs, ơ_C_ denotes standard deviation of costs, µ_E_ denotes mean effects, ơ_E_ denotes standard deviation of effects; normal distributions for costs and effects are assumed; correlation between costs and effects of each program is denoted by ρ; QALY denotes quality-adjusted life-years.

**Fig. 1:**
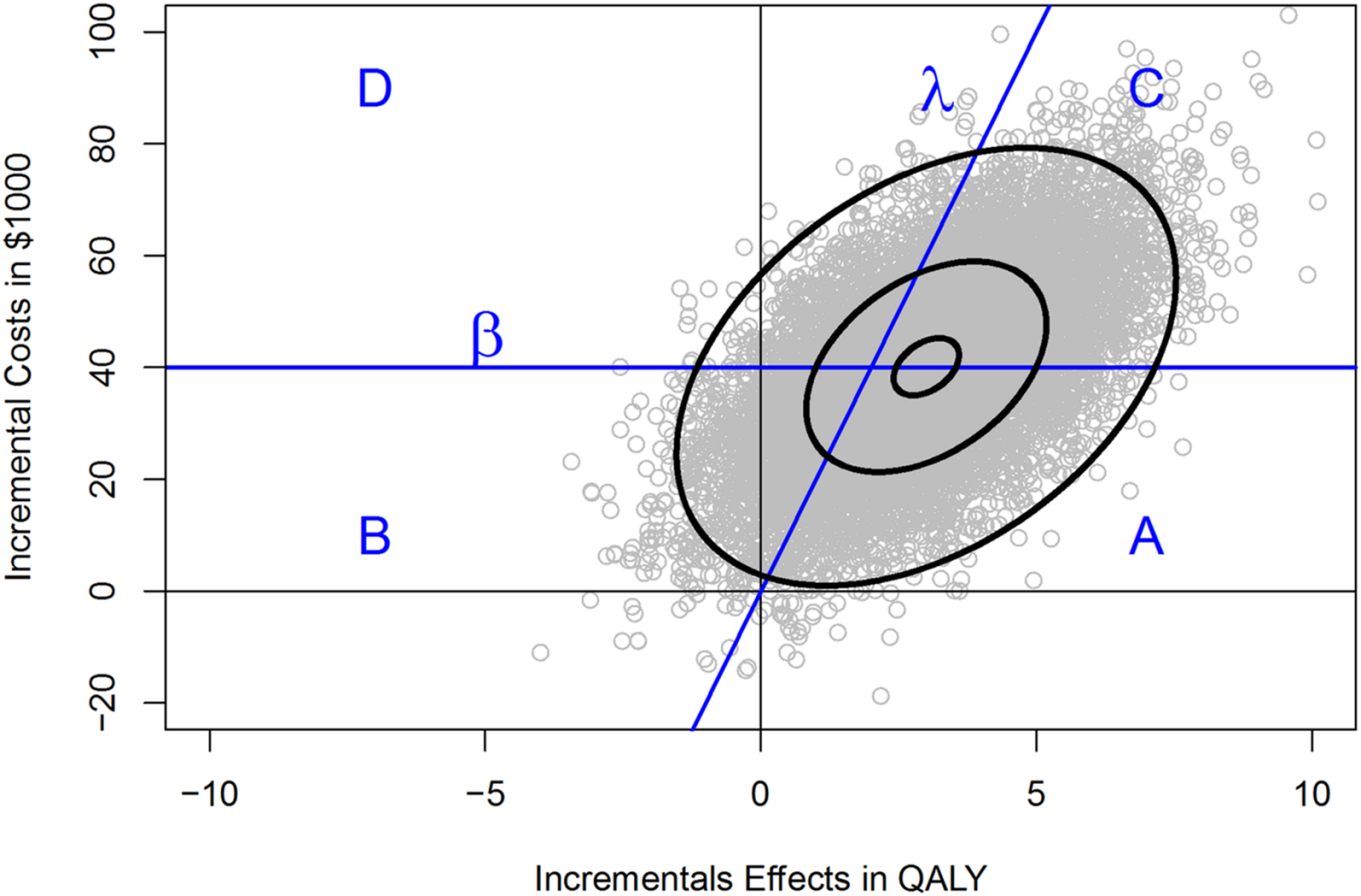
Joint distribution of incremental costs and effects on the cost-effectiveness plane. λ denotes the threshold cost-effectiveness ratio (maximum WTP per QALY), β denotes the budget constraint. In area A the intervention is both affordable and cost-effective, in area B the intervention is affordable but cost-ineffective, in area C the intervention is cost-effective but not affordable, in area D the intervention is neither affordable nor cost-effective

 The expected incremental cost-effectiveness ratio is hence $40,000 ($91,000-$51,000) divided by 3 QALYs (14 − 11 QALYs), that is $13,333 per QALY gained. By sampling 10,000 times from the four distributions listed above we can estimate a joint distribution for incremental costs and effects, that is typically displayed on the cost-effectiveness plane as shown in Fig. 1. The joint distribution of incremental costs and effects is separated by the ceiling ration λ into an area that is cost-effective (below the ceiling ratio line) and that is not cost-effective (above the ceiling ratio line) [16, 24]. Likewise, the joint distribution can be divided into an affordable area below the budget line β and an unaffordable area above the budget line. Therefore, these two threshold lines (λ and β) separate the joint distribution of incremental costs and effects into four areas:

1. area A where the intervention is both affordable and cost-effective.
2. area B where the intervention is affordable but not cost-effective.
3. area C where the intervention is not affordable but cost-effective.
4. area D where the intervention is not affordable and not cost-effective.

**Fig. 2:**
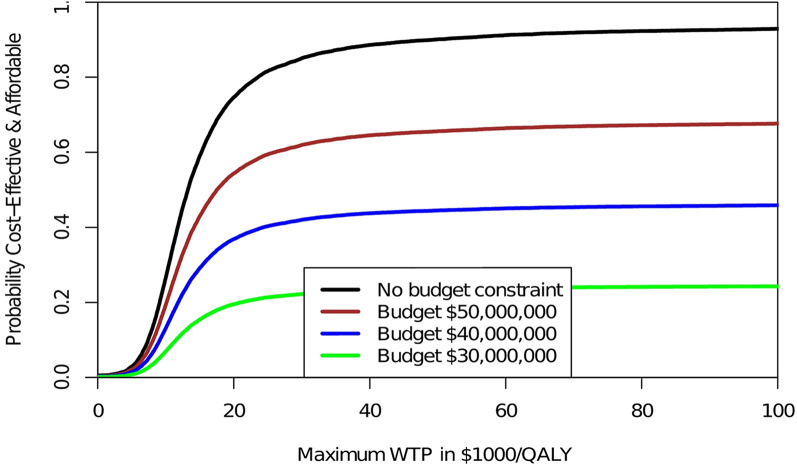
Cost-effectiveness affordability curves (CEAFCs). Comparing different budget constraints of program F versus program E. The CEAC is depicted by the black curve without any budget constraint

Area A is therefore of interest to most decision makers that need to assure that the intervention (program F versus E) is cost-effective (i.e., with an acceptable return on investment) and within the available budget. We can now construct different CEAFCs for each budget constraint as shown in Fig.  2. In Fig.  2 the CEAFCs are constructed assuming 1000 patients and a budget constraint of $30,000,000, $40,000,000 and $50,000,000, respectively. In the absence of a budget constraint, the CEAFC corresponds to the CEAC. However, as most decision makers need to contain costs and stay within a given budget, the joint probability that the intervention is both affordable and cost-effective (i.e., area A in Fig. 1) is of most interest. Even for individual patient decision-making with uncertain cost outcomes, the cost-effectiveness affordability curve helps to better clarify the likelihood of the intervention being both cost-effective (i.e., below the decision maker’s maximum WTP per health gain) and affordable (i.e., below his available budget). If only budget impact, i.e. affordability, would be of interest, we could plot the affordable proportion of the cost distribution as a function of the budget line β.

**Fig. 3:**
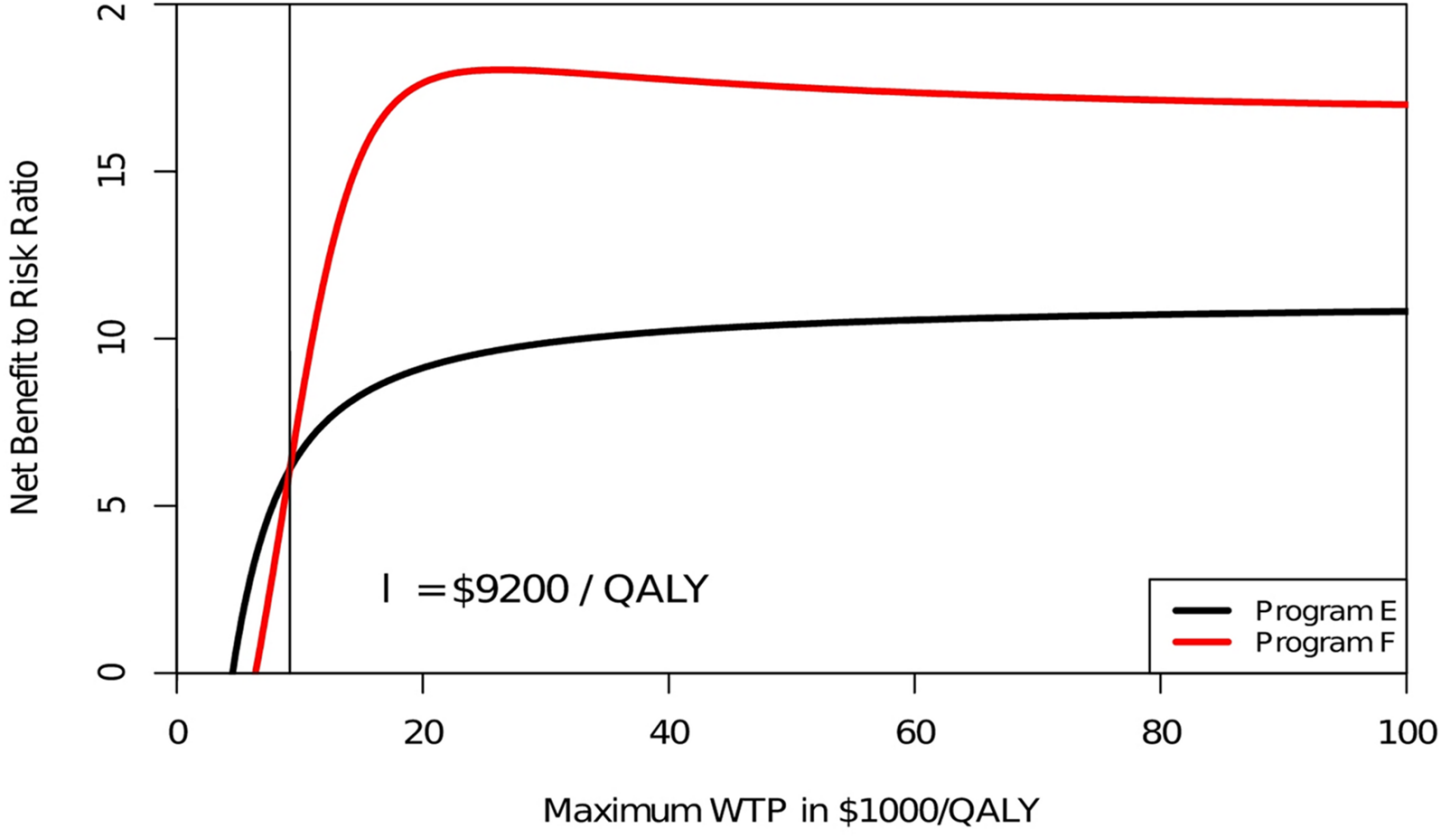
Cost-effectiveness risk-aversion curve (CERAC). Program E offers a higher expected net benefit per unit of downside risk than program F up to a ceiling ratio of $9200 per QALY. Program F becomes preferable for ceiling ratios higher than $9200 per QALY

In addition, decision makers are usually not risk neutral and have an implied attitude towards risk. It has been shown that for health outcomes and large investments, decision makers have a risk averse attitude [22, 25, 26, 28]. The CERAC addresses this issue by borrowing techniques used for risk-adjusted performance measures in finance, in particular the Sortino ratio, without the need to elicit an explicit preference function [23, 24, 33]. The methodology of the CERAC has previously been described and discussed in the literature [23, 24, 26, 33]. Here, we formally show how it is constructed using hypothetical data (see Table 1) from a probabilistic cost-effectiveness analysis [23, 24]. For each ceiling ratio λ we can construct a net benefit to risk ratio *S*_*NMB*_ as follows

1 $$\:\begin{array}{c}{S}_{NMB}=\frac{{\mu\:}_{NMB}}{{DD}_{NMB}}\end{array}$$

where

2 $$\:\begin{array}{c}{\mu\:}_{NMB}=\:{\mu\:}_{E}\:\:.\lambda\:-{\mu\:}_{C}\:\end{array}$$

where *µ*_*NMB*_ denotes the expected Net Monetary Benefit (NMB) of a program, *µ*_*E*_ denotes mean effect, *µ*_*C*_ mean cost of a program, and λ the ceiling ratio. *DD*_*NMB*_ denotes the downside deviation, defined as

3 $$\:\begin{array}{c}{DD}_{NMB}=\sqrt{\frac{1}{n}\sum\:_{i=1}^{n}{\left({NMB}_{i}-{\mu\:}_{NMB}\right)}^{2}f\left(t\right)\:\:}\end{array}$$

$$\:f\left(t\right)=1\:if\:{NMB}_{i}<{\mu\:}_{NMB}$$

$$\:f\left(t\right)=0\:if\:{NMB}_{i}\ge\:{\mu\:}_{NMB}$$

where *NMB*_*i*_ reflects a sample observation. The *DD*_*NMB*_ reflects the root-mean square of all sample observations. The *S*_*NMB*_ (Eq.  1) penalizes the expected NMB of a program µ_NMB_ for its “bad” risk as reflected in *DD*_*NMB*_, i.e. below average returns. For a more detailed discussion we refer to Sendi [23] and Sendi et al. [24, 33]. The CERAC can be calculated for each program and the expected net monetary benefit to risk ratios can then be compared. A higher net monetary benefit to risk ratio indicates more expected return per unit of “bad” risk and should therefore be preferred by a risk averse decision maker. For the hypothetical example shown in Table  1, the CERACs are calculated and depicted in Fig.  3. As can be seen, for a ceiling ratio below $9200 per QALY, program E is the preferred strategy, while for ceiling ratios above $9200 program F offers more expected return per unit of “bad” (i.e., downside) risk.

## Results

A total of 7303 records were initially identified through database searches (Figure S1); 919 remained after removal of duplicates for title and abstract screening. A total of 395 articles were included for full-text screening from database searches and 37 articles from citation searches. Of these 395 studies, 57 remained for qualitative analysis (Figure S1, supplementary file). Figure S1 (supplementary file) displays the PRISMA 2020 (Preferred Reporting Items for Systematic Reviews and MetaAnalyses) flow diagram. Table 2 displays the descriptive characteristics of the studies included in this study with respect to sensitivity analysis and handling uncertainty. Table S1 (supplementary file) displays content and further design characteristics of the included studies.

**Table 2:** Cost-effectiveness analyses in dental medicine included in this review

Author Publication Year Reference	Country	Intervention	Study design	Deterministic SA Yes/No	Probabilistic SA Yes/No	Budget Impact Yes/No	Risk Aversion Yes/No
Ahn et al. [46], 2022	South Korea	Early dental visits of children to prevent childhood caries	Clinical Trial	Yes	No	No	No
Anopa et al. [47], 2023	UK	Preventive value of fluoride varnish in nursery schools	Clinical Trial	Yes	Yes	No	No
Björksved et al. [48], 2021	Sweden	Open and closed surgical for correction of palatally displaced canines	Clinical Trial	No	Yes	No	No
Boachie et al. [49], 2023	South Africa	Dental caries prevention strategies in schools	Modeling Study	No	Yes	No	No
Martins et al. [50], 2023	Germany	Full versus selective root canal retreatment	Modeling Study	Yes	Yes	No	No
Cronin et al. [51], 2021	Ireland	Water fluoridation	Modeling Study	Yes	Yes	Yes	No
Davidson et al. [52], 2022	Sweden	Dental-Health FRAMM Guideline for Caries Prevention	Modeling Study	Yes	No	No	No
Da Costa Rosa et al. [53] ., 2024	Brasil	Restorative treatments for permanent molars with severe molar incisor hypomineralization	Modeling Study	Yes	Yes	Yes	No
Da Silva et al. [54] , 2025	Brasil	Atraumatic restorative treatment with and without chemical-mechanical caries removal agents based on pain and time for selective removal of carious tissue	Modeling Study	No	Yes	No	No
Effenberger et al. [55], 2022	South Africa	Fluoride varnish for caries prevention	Clinical Trial	No	Yes	No	No
Egil et al. [56], 2022	Turkey	Fissure sealants for caries prevention	Modeling Study	Yes	No	No	No
Fueki et al. [57], 2021	Japan	Thermoplastic resin dentures	Clinical Trial	Yes	No	No	No
Halasa-Rappel et al. [58], 2021	USA	Pit-and-fissure sealants on primary molars	Cohort Study	Yes	No	Yes	No
Hansson et al. [59] , 2024	Sweden	Posterior crossbite corrections in the early mixed dentition with quad helix or rapid maxillary expander	Clinical Trial	Yes	Yes	No	No
Herdman et al. [60], 2022	UK	Cost-effectiveness model for denture cleaning strategies	Modeling Study	Yes	No	No	No
Janusz et al. [61] , 2023	USA	Population-Level Dental Caries Prevention Strategies in US Children	Modeling Study	Yes	No	No	No
Jevdjevic et al. [62] 2021	Germany	Food package labeling to reduce caries	Modeling Study	Yes	Yes	No	No
Kachapila et al. [63], 2021	UK	Preoperative chlorhexidine mouthwash at reducing postoperative pneumonia among abdominal surgery patients	Modeling Study	Yes	Yes	No	No
Kanzow et al. [64], 2021	Germany	Restoration repair versus replacement	Clinical Trial	Yes	Yes	No	No
Katsura et al. [65], 2021	Japan	Measures against metallic dental restorations for head and neck radiotherapy	Combined	No	No	No	No
Liu et al. [66], 2021	China	Sealing with resin or ART sealant	Combined	Yes	Yes	No	No
Lopes Martins et al. [67], 2021	Brazil	Photobiomodulation therapy for prevention of radiotherapy-induced severe oral mucositis	Clinical Trial	Yes	No	No	No
Losenická et al. [68] 2021	Czech Republic	Three-unit fixed dental prosthesis versus implant	Modeling Study	Yes	No	No	No
Merchan et al. [69], 2022	Brazil	Different endodontic instrumentation techniques	Clinical Trial	No	No	No	No
Nantanee and Sriratanaban [70] 2023	Thailand	Benefits of a fluoride varnish application program during well-child visits by 9-to 30-month-old children in three areas	Modeling Study	Yes	Yes	No	No
Naved et al. [71] , 2024a	USA	Irreversible pulpitis in mature permanent Teeth, pulpotomy vs. root canal treatment	Modelling Study	Yes	Yes	No	No
Naved et al. [72] , 2024b	USA	Restoration of endodontically treated teeth	Modelling Study	Yes	Yes	No	No
Naved et al. [73], 2023	Canada	Regenerative Endodontics versus MTA Apexification	Modeling Study	Yes	Yes	No	No
Nguyen et al. [74], 2023	Australia	Modeled health economic and equity impact on dental caries and health outcomes from a 20% sugar sweetened beverages tax	Modeling Study	Yes	Yes	Yes	No
Nogueira et al. [75] , 2021	Brazil	Loaded single-implant mandibular overdentures	Clinical Trial	Yes	No	No	No
Okubo et al. [76], 2023	Japan	Professional and mechanical oral care for preventing pneumonia in nursing home residents	Modeling Study	Yes	Yes	No	No
Otake et al. [77], 2022	Japan	Milled complete dentures	Clinical Trial	Yes	No	No	No
Pires et al. [78], 2021	Brazil	Glass fiber post and direct composite compared to other restorations	Clinical Trial	Yes	No	No	No
Rodriguez et al. [79], 2022	Chile	Probiotics and fluoride varnish in caries prevention	Clinical Trial	No	Yes	No	No
Savolainen et al. [80] 2023	Sweden	Partial versus stepwise caries removal of deep caries lesions	Modeling Study	Yes	No	No	No
Sanghvi et al. [81], 2023	UK	Removal of compromised first permanent molars	Modeling Study	Yes	No	No	No
Santos et al. [82] , 2024	Brasil and USA	At-home bleaching versus whitening toothpastes for treatment of tooth discoloration	Modeling Study	No	Yes	No	No
Savolaninen et al. [83] , 2025	Sweden	Root canal treatment and implant-supported crowns	Modeling Study	Yes	No	No	No
Schwendicke et al. [84] 2021	Germany	Proximal caries detection on bitewing radiographs with artificial intelligence	Modeling Study	Yes	Yes	No	No
Schwendicke et al. [85] 2021	Germany	Glass hybrid versus composite in permanent molars	Modeling Study	Yes	Yes	No	No
Schwendicke et al. [86] 2022	Germany	Caries detection on bitewing radiographs with artificial intelligence	Modeling Study	Yes	Yes	No	No
Schwendicke et al. [87] 2022	Germany	Artificial intelligence for caries detection	Modeling Study	Yes	Yes	No	No
Schwendicke et al. [88] 2023	Germany	School-based caries screening using transillumination	Modeling Study	Yes	Yes	No	No
Sharda et al. [89] , 2025	India	School-based toothbrushing program	Modeling Study	Yes	Yes	No	No
Sobral et al. [90], 2022	Brasil	Photobiomodulation in oral lichen planus patients	Clinical Trial	Yes	No	No	No
Souto et al. [91], 2021	Brasil	Smoking cessation to avoid tooth loss	Modeling Study	Yes	Yes	No	No
Tan et al. [92] , 2025	Singapore	Silver diamine fluoride, NaF varnish, direct restorations	Modeling Study	Yes	Yes	No	No
Tang et al. [93] , 2024	China	Fluoride varnish application for caries prevention	Modeling Study	Yes	Yes	No	No
Tannous et al. [94], 2021	Australia	Midwifery-initiated oral health service	Combined	Yes	No	No	No
Tekpmar et al. [95] , 2024	Turkey	Implant-supported crowns vs. tooth-supported fixed dental prostheses	Modelling study	Yes	Yes	No	No
Tonmukayakul et al. [96]2021	Australia	Atraumatic restorative treatment to manage early childhood caries	Modeling Study	Yes	Yes	No	No
Tonmukayakul et al. [97] , 2025	Australia	Atraumatic restorative treatment	Modeling Study	Yes	Yes	No	No
Victory et al. [34], 2022	UK	Talk intervention to prevent caries reoccurrence	Modeling Study	Yes	Yes	Yes	Yes
Zang et al. [98], 2023	China	Nonsurgical root canal treatment versus single-tooth implant	Cohort Study	Yes	No	No	No
Zhu et al. [99] , 2024	China	Fluoride varnish for root caries prevention	Modeling Study	Yes	Yes	No	No
Zhurakivska et al. [100],2022	Italy	Antibiotic prophylaxis at dental implant placement	Modeling Study	Yes	Yes	No	No
Zhurakivska et al. [101], 2023	Italy	Treatment options for the rehabilitation of the total edentulous mandible	Modeling Study	Yes	Yes	No	No

From a total of 57 published cost-effectiveness analyses, 49 (85%) used a deterministic sensitivity analysis and 37 (65%) used a probabilistic sensitivity analysis (Table 2). Budget impact was considered in 5 articles (9%) but only the paper by Victory et al. [34] made use of the CEAFC (Table 2). Likewise, only Victory et al. [34] formally applied the CERAC in their economic evaluation. The use of more advanced econometric methods such as the CEAFC and CERAC is still limited in dental medicine.

Further study characteristics are shown in more detail in Table S1 (supplementary file). Of the 57 studies included in this systematic review, most used a Markov model (22/57) or a decision tree model (15/57) (Table S1, supplementary file, with references). Cost-effectiveness analyses alongside clinical trials or based on observational studies were also often reported (15/57), see Table S1 (supplementary file, with references). QALYs were reported as outcome in 6/57 studies and the remainder used dental specific outcomes, which were very heterogenous (Table S1, supplementary file, with references). Details with respect to time horizon, perspective and discount rate are also shown in Table S1 (supplementary file). The results of the CHEERS 2022 checklist are reported in detail in Table S2 (supplementary file). The average compliance in reporting was 81%.

## Discussion

In the present paper we have systematically reviewed cost-effectiveness analyses in dental medicine that were published since 2021. The number of published cost-effectiveness analyses in dentistry is increasing, and using a cut-off year of 2021 accounts for the fact that more recent methodological developments in handling uncertainty—particularly CERAC—may be captured. [23, 26, 33]. We were able to find 57 cost-effectiveness analyses (see Table 2) and we did not exclude cost-minimisation analyses, as this is a special case of cost-effectiveness analysis where the same issues with respect to handling uncertainty are of importance. Most articles used deterministic and probabilistic sensitivity analysis to address uncertainty surrounding costs and effects, and ultimately the cost-effectiveness of the intervention. These methods are well established, and we therefore do not discuss these in more detail in the present paper. The use of methods to address uncertainty with respect to affordability, which addresses budget constraints, and risk-aversion, however, is limited. Only one paper made use of the CEAFC and CERAC [34]. A possible explanation may be that in most countries, dentistry is not covered by compulsory health insurance. But we feel that this information is nonetheless also relevant for decisions at the patient level, as projected discounted costs may need to meet the available budget of the patient. In addition, patients may exhibit different levels of risk-aversion with respect to their oral health and are usually not risk-neutral. In countries with a public dental healthcare system, risk-aversion may also be relevant to policymakers who intend to design/reform a national/public dental insurance system.

The CEAFC and CERAC are additional helpful tools to provide valuable information for decision-makers [16, 23, 24]. The CEAC alone as a source of information may be misleading, since the CEAC does not inform about the size of a program [35]. In addition, different joint distributions of incremental costs and effects may lead to the same CEAC, as the CEAC does not capture radial shifts in the North-East quadrant of the cost-effectiveness plane [16, 35]. On the other hand, these limitations are addressed by the CEAFC. The CEAFC has been used in cost-effectiveness analyses with pressing budget constraints in medicine [36–40] such as in developing countries but also in dentistry [34, 41]. Of note, the ceiling ratio in the North-East quadrant of the cost-effectiveness plane reflects the decision maker’s maximum WTP for health gain, whereas in the South-West quadrant of the cost-effectiveness plane it reflects the decision-maker’s minimum willingness to accept (WTA) to forgo health. In a previous study we have shown that there is usually a WTP-WTA disparity for the same health condition, and this disparity may be captured and illustrated in the CEAC [1, 42]. Other approaches to address affordability concerns have been suggested in the literature [43]. For example Lomas [43]suggested to adjust the ceiling ratio as a function of the available budget and health opportunity cost which formally includes budget impact analysis into cost-effectiveness analysis. Rather than estimating the joint probability that an intervention is both cost-effective and affordable, affordability directly impacts the ceiling ratio used as a cut-off point used for resource allocation [43].

Risk aversion is an important additional issue surrounding uncertainty of costs and effects, since decision makers are usually risk averse towards great losses. Health is regarded as the most valuable good, therefore it is natural to be risk averse regarding health issues [29]. Furthermore, health is not a transferable good, that is, those who gain health cannot share their improved health state with those who loose health, which further stresses the importance of risk-aversion [29]. Incorporating risk-aversion into cost-effectiveness analysis may be complicated by the fact that usually a risk-aversion parameter that is part of a preference function over uncertain costs and effects may need to be elicited from the decision maker [25, 26, 28, 44]. This is not an easy task, and many decision makers may not want to explicitly exhibit their risk-preferences to define a utility function over uncertain costs and effects [22]. The CERAC simplifies this task as risk-aversion is implied in the risk-adjusted performance measures commonly used in finance [23] and there is no need for an explicit preference function. There are a number of different risk-adjusted performance measures in finance, but the one suggested for constructing the CERAC is based on the Sortino ratio, which rather assumes a higher level of risk-aversion [26, 31]. The expected NMB of a program is divided by its downside deviation as defined above and this metric can be calculated for each ceiling ratio, which then allows to construct the CERAC. It should be noted that the CERAC is not a fixed metric and that depending on the decision maker, his minimum acceptable return for a program that defines downside deviation can be varied, which then leads to a different CERAC as detailed elsewhere [24, 33]. The CERAC, although recently introduced, has been used by several studies in both medicine and dentistry [24, 34, 40, 45].

This review shows that while more recent methods for addressing uncertainty in cost-effectiveness analysis—particularly regarding budget constraints (i.e., affordability) and risk aversion—are being used, their application remains limited. To support better decision-making in dental health care, it is recommended to conduct additional analyses, such as estimating the CEAFC and CERAC alongside the CEAC in probabilistic cost-effectiveness analysis. More empirical studies are needed to determine whether the additional information provided by the CEAFC and CERAC is useful for real-world decision-making.

## Electronic supplementary material

Below is the link to the electronic supplementary material.

Supplementary Material 1

Supplementary Material 2

Supplementary Material 3

Supplementary Material 4

## Data Availability

No datasets were generated or analysed during the current study.
