# Supplementary material for: Handling uncertainty in cost-effectiveness analysis in dental medicine: a systematic review with a focus on affordability and risk-aversion
Source: Cost Eff Resour Alloc. 2025 Jun 19;23:32. doi: 10.1186/s12962-025-00641-9 (PMC12180185; doi:10.1186/s12962-025-00641-9)
Supplement: Supplementary file 1 — Supplementary Material 1 [file 12962_2025_641_MOESM1_ESM.docx]

Handling uncertainty in economic evaluation in dental medicine: a systematic review with a focus on affordability and risk-aversion

**Search Strategy**

Search was conducted on Apr 18, 2025

Filters:

Language

- English
- German

Publication Date after January 1st, 2021

**PubMed Search Strategy**

| # | Search | Results |
| --- | --- | --- |
| 1 | Cost-effectiveness | 36359 |
| 2 | Economic evaluation | 26309 |
| 3 | Incremental cost-effectiveness ratio | 5974 |
| 4 | ICER | 3216 |
| 5 | Cost utility | 77758 |
| 6 | Cost-Benefit Analysis (MeSH) | 16935 |
| 7* | 1 OR 2 OR 3 OR 4 OR 5 OR 6 | 117504 |
| 8 | Tooth | 48625 |
| 9 | Dent | 38465 |
| 10 | Dental | 141457 |
| 11 | Dentistry (MeSH) | 151391 |
| 12* | 8 OR 9 OR 10 OR 11 | 225766 |
| 13 | 7 and 12 | 3413 |

*Preceding search criteria are combined

**Web of Science Search Strategy:**

| # | Search | Results |
| --- | --- | --- |
| 1 | Cost-effectiveness | 44741 |
| 2 | Economic evaluation | 46486 |
| 3 | Incremental cost-effectiveness ratio | 4889 |
| 4 | ICER | 4250 |
| 5 | Cost utility | 20082 |
| 6 | Cost-Benefit Analysis | 6439 |
| 7* | 1 OR 2 OR 3 OR 4 OR 5 OR 6 | 108011 |
| 8* | Dentistry (Topic) | 17129 |
| 9 | 7 and 8 | 162 |

*Preceding search criteria are combined

**Cochrane Search Strategy**

| # | Search | Results |
| --- | --- | --- |
| 1 | Cost-effectiveness | 9108 |
| 2 | Economic evaluation | 3383 |
| 3 | Incremental cost-effectiveness ratio | 1519 |
| 4 | ICER | 1074 |
| 5 | Cost utility | 2049 |
| 6 | Economic | 8907 |
| 7 | Cost | 23092 |
| 8 | Cost-Benefit Analysis (MeSH) | 2783 |
| 9* | 1 OR 2 OR 3 OR 4 OR 5 OR 6 OR 7 OR 8 | 27883 |
| 10 | Tooth | 13575 |
| 11 | Dent | 399 |
| 12 | Dental | 13619 |
| 13 | Dentistry (MeSH) | 5162 |
| 14* | 10 OR 11 OR 12 OR 13 | 21432 |
| 15 | 9 and 14 | 866 |

*Preceding search criteria are combined

**Embase Search Strategy**

| # | Search | Results |
| --- | --- | --- |
| 1 | Cost-effectiveness | 59266 |
| 2 | Economic evaluation | 9591 |
| 3 | Incremental cost-effectiveness ratio | 5323 |
| 4 | ICER | 5570 |
| 5 | Cost utility | 4494 |
| 6 | Cost-Benefit Analysis (MeSH) | 15457 |
| 7* | 1 OR 2 OR 3 OR 4 OR 5 OR 6 | 75369 |
| 8 | Tooth | 69545 |
| 9 | Dent | 35685 |
| 10 | Dental | 168088 |
| 11 | Dentistry (MeSH) | 149130 |
| 12* | 8 OR 9 OR 10 OR 11 | 260852 |
| 13 | 7 and 12 | 2048 |

*Preceding search criteria are combined

**Econlit Search Strategy**

| # | Search | Results |
| --- | --- | --- |
| 1 | Cost-effectiveness | 34940 |
| 2 | Economic evaluation | 10185 |
| 3 | Incremental cost-effectiveness ratio | 5152 |
| 4 | ICER | 3850 |
| 5 | Cost utility | 5080 |
| 6 | Cost-Benefit Analysis (MeSHdent) | 19833 |
| 7* | 1 OR 2 OR 3 OR 4 OR 5 OR 6 | 48564 |
| 8 | Tooth | 51120 |
| 9 | Dent | 39501 |
| 10 | Dental | 163149 |
| 11 | Dentistry (MeSH) | 143617 |
| 12* | 8 OR 9 OR 10 OR 11 | 247971 |
| 13 | 7 and 13 | 814 |

*Preceding search criteria are combined
