# Supplementary figures and images for: Handling uncertainty in cost-effectiveness analysis in dental medicine: a systematic review with a focus on affordability and risk-aversion
Source: Cost Eff Resour Alloc. 2025 Jun 19;23:32. doi: 10.1186/s12962-025-00641-9 (PMC12180185; doi:10.1186/s12962-025-00641-9)

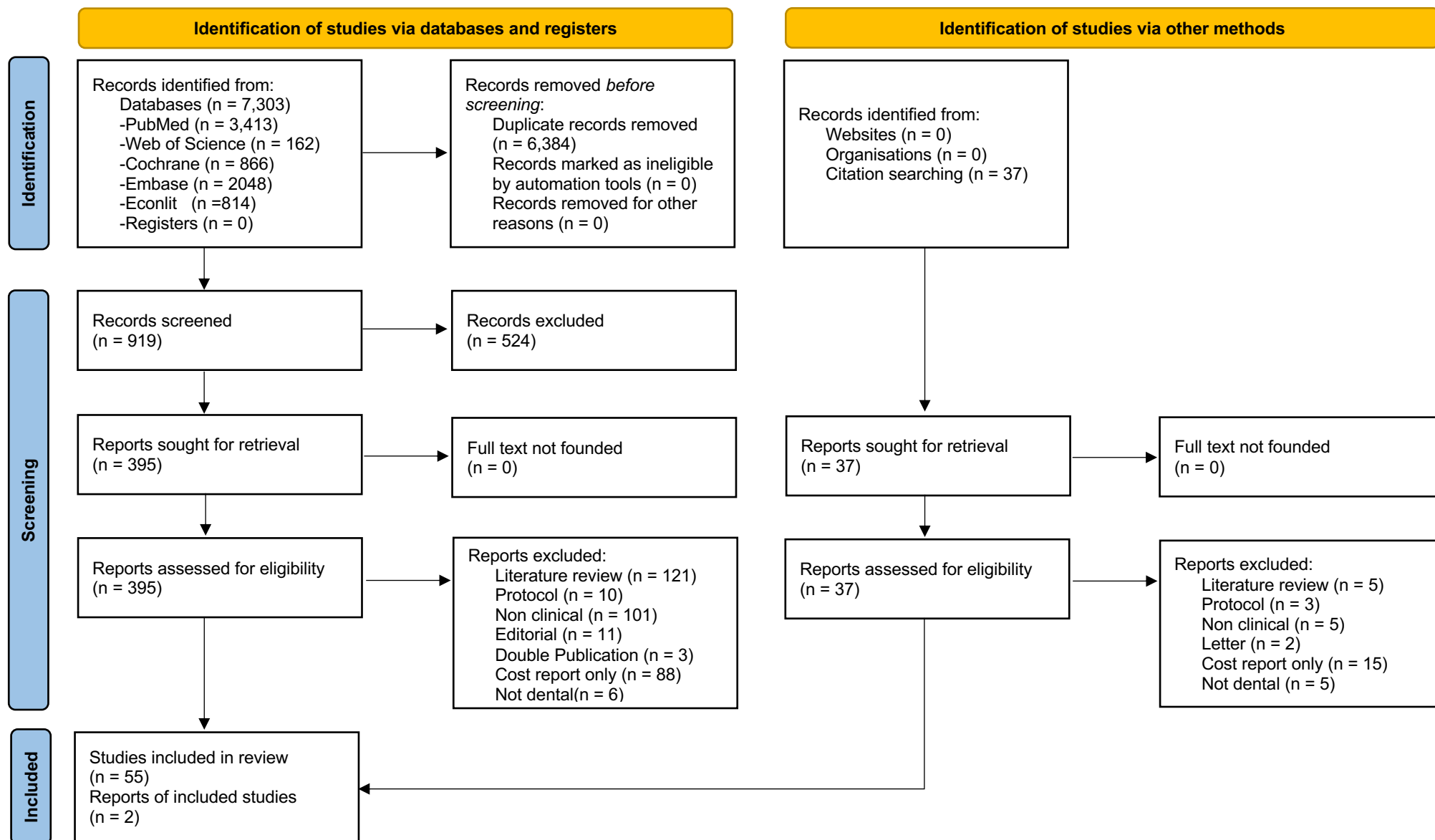

Supplement: Supplementary file 2 — Supplementary Material 2 [file 12962_2025_641_MOESM2_ESM.pdf]
